# Distribution of pseudocysts in *Fascioloides magna* infected livers of red deer and fallow deer

**DOI:** 10.2478/helm-2025-0015

**Published:** 2025-09-30

**Authors:** D. Konjević, P. Verzak, K. Jerabek, M. Bujanić, Z. Janicki

**Affiliations:** 1 University of Zagreb, Faculty of Veterinary Medicine, Heinzelova 55, 10000 Zagreb, Croatia; 2 Rijeka Veterinary Station, Buje clinic, Digitronska 8, 52460 Buje Croatia; 3 EWOPHARMA d.o.o., Jadranska avenija 9, 10000 Zagreb Croatia

**Keywords:** red deer, fallow deer, *Fascioloides magna*, pseudocyst localization

## Abstract

In Europe, red deer and fallow deer are the defi nitive hosts for the non-native fl uke *Fascioloides magna*. Upon entering the host, a juvenile fl uke emerges from the metacercariae and travels through the abdominal cavity in search of the liver. The aim of this study was to determine the distribution of *F. magna* pseudocysts in the livers of red deer and fallow deer, and to investigate whether this distribution varies within the livers according to host species and the severity of infection. In this study, 143 red deer livers and 178 fallow deer livers were collected. Livers were divided into three sections (observed from the diaphragm side): left containing the lobus hepatis sinister, middle containing the lobus caudatus and lobus quadratus, and right one containing the lobus hepatis dexter. Sections were sliced to a 2 cm thickness and analysed. A signifi cantly higher number of pseudocysts was found in the middle section of the liver compared to the left or right sections, while no differences were found between the left and right sections. The odds ratio indicates a 7 times (red deer) and 3.7 times (fallow deer) higher probability of pseudocysts being present in the middle section compared to the left section, or 10 and 3.8 times, respectively, compared to the right section. Red deer livers generally harboured more pseudocysts than fallow deer livers in all sections. Both species exhibited a similar pattern of pseudocyst distribution in the liver, with the middle section appearing to be the most suitable site for pseudocyst localization due to the abundance of blood vessels and bile duct terminations. No relationship was found between the severity of infection and pseudocyst distribution.

## Introduction

The Digenean trematode *Fascioloides magna* was fi rst described in an infected wapiti deer (*Cervus elaphus canadensis*) from Royal Park La Mandria near Turin, Italy (Bassi, 1875). According to Králová-Hromadová *et al*. (2011), this parasite was introduced to Europe at least twice, resulting in the formation of three permanent foci of infection: in Italy, the Czech Republic and the Danube region. As a non-native parasite in Europe, *F. magna* established associations with new intermediate (mainly *Lymnaea truncatula*) and fi nal hosts (see Králová-Hromadová *et al*., 2016). Though the terminology varies in the literature, wild fi nal hosts in Europe can be divided into defi nitive (red deer, *Cervus elaphus*, and fallow deer, *Dama dama*), aberrant (roe deer, *Capreolus capreolus*, moufl on, *Ovis musimon*, experimentally chamois, *Rupicapra rupicapra*, and accidentally Alpine ibex, *Capra ibex*), and dead-end (wild boar, *Sus scrofa*) (Králová-Hromadová *et al*., 2016; Bujanić *et al*., 2022). The course of infection and clinical picture depend on the host type.

Upon ingestion by the final host, the juvenile fluke emerges from the metacercariae in the intestines. It penetrates the intestinal wall and migrates to the liver (Králová-Hromadová *et al*., 2016). Occasionally, in aberrant hosts, the juvenile fluke can accidentally migrate to other organs, such as the spleen, lungs, or kidneys (Stiles *et al*., 2021). In those cases, the fluke must enter the organ to recognize it (Sukhdeo *et al*., 1987), and only then, if the host survives, can it leave the organ to continue its search for the liver. However, such a migration error can lead to excessive haemorrhaging, infection, inflammation and even death of the host (Stiles *et al*., 2021). If the fluke succeeds in its search for the liver, it will penetrate Glisson’s capsule and start migrating through the liver parenchyma (Králová-Hromadová *et al*., 2016). This behavior causes excessive damage to the liver parenchyma and substantial hemorrhaging, although the severity is host-dependent. This damage is visible as bloody channels or haematoma. Acute damage to the liver is only stopped by the formation of a pseudocyst in which the fluke matures and starts to produce a high number of eggs daily.

Despite the great interest that *F. magna* has aroused in the scientific community, there are no studies on the distribution of pseudocysts in the livers of infected animals. In this paper, we present the distribution of pseudocysts within the livers of definitive hosts, red deer and fallow deer, and hypothesize that pseudocyst distribution could serve as a criterion for grading the infection.

## Material and Methods

### Sampling

A total of 321 livers were collected for this study, 143 from red deer and 178 from fallow deer. Samples were collected from hunters following the regular implementation of Game Management Plans. Therefore, the sampling type used was non-probability, convenience sampling. Red deer samples originated from open hunting grounds in three Croatian counties: Bjelovar-Bilogora County, Sisak-Moslavina County and Vukovar-Srijem County. Fallow deer samples originated from breeding areas in Kunjevci (Vukovar-Srijem County) and Poljodar (Bjelovar-Bilogora County), both of which are large fenced areas. Following evisceration, livers were stored in plastic bags, labelled and frozen at approximately -20 °C until analysis. Samples were collected within the framework of a Croatian Science Foundation Grant (IP-8963).

### Liver analysis

**Fig. 1.**
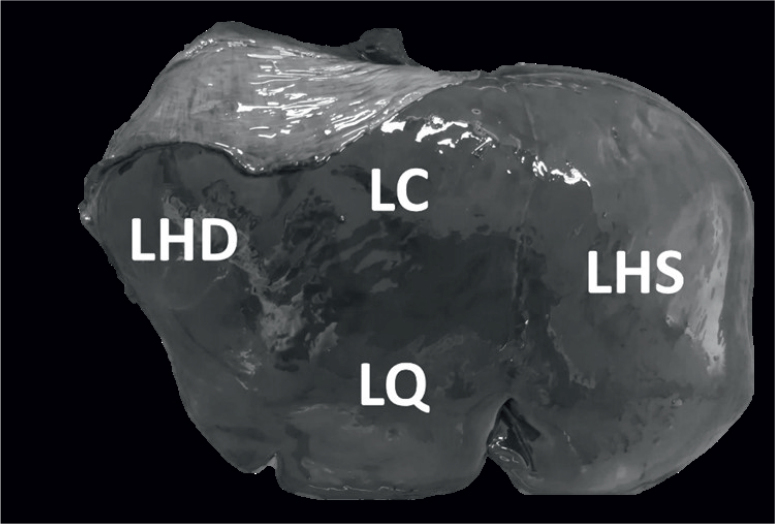
Division of livers into middle (*lobus caudatus* LC, *lobus quadratus* LQ), left (*lobus hepatis sinister* LHS) and right (*lobus hepatis dexter* LHD) sections.

Externally, livers were analysed for surface irregularities, enlargement, fibrin deposits, loss of translucency of the Glisson’s capsule and traces of iron-porphyrin. Each liver was sliced into 2 cm-thick slices and analysed for any developmental stage of *F. magna* and the presence of pseudocysts (degrading pseudocysts were also recorded). Pseudocyst presence was noted based on the location in the liver. From the diaphragm side, livers were divided into three sections: left, composed of the left hepatic lobe *(lobus hepatis sinister* - LHS), middle, composed of the quadrate and caudate lobes (*lobus quadratus* – LQ; *lobus caudatus* -LC), and right, containing the right hepatic lobe (*lobus hepatis dexter* -LHD) (Fig. 1).

### Data analysis

Data were analyzed using descriptive statistics, including determination of the average, maximum, and minimum values, as well as standard deviation. Statistical significance was calculated using the χ2 test with Yates’ correction. Significance was set at p<0.05. The odds ratio was calculated according to the following formula: OR = a × d/(b × c), using a 2 × 2 Table. The confidence level was set at 95 % and calculated according to the formula: 95 % CI = ex-p(In(OR) – 1.96 × SE{In(OR)}) to exp(In(OR) + 1.96 × SE{In(OR)}).

## Ethical Approval and/or Informed Consent

The study was approved by the Ethics Committee of the University of Zagreb, Faculty of Veterinary Medicine (No. 640-01/18-17/60 of 9 September 2018).

## Results

### Red deer

In the case of red deer, only infected livers were analysed. Table 1 presents the findings of pseudocysts in the analysed livers of red deer. The highest average number of pseudocysts was found in the middle section (n=655; $\bar{\text{x}}=4.58$; min=0; max=56; SD=6.35), followed by left (n=321; $\bar{\text{x}}=2.24$; min=0; max=15; SD=3.0) and right sections (n=277; $\bar{\text{x}}=1.93$; min=0; max=12; SD=2.52). The majority of pseudocysts (73.03 %) were found in the middle section, followed by 16.45 % in the left and 10.53 % in the right sections. In 22.4 % of livers, pseudocysts were found exclusively in the middle part of the liver.

**Table 1.** Descriptive statistics of pseudocyst localization in the liver of red deer and fallow deer.

pseudocysts	Red deer (n=143)			Fallow deer (n=178)		
	Left	Middle	Right	Left	Middle	Right
Total number	321	655	277	42	95	41
$\bar{\text{x}}$	2.24	4.58	1.93	0.6	1.3571	0.5857
SD	3.00	6.35	2.52	0.8236	1.3835	1.1098
Min	0	0	0	0	0	0
Max	15	56	12	3	8	5

Table 2 presents a comparison between the middle section and the left and right sections of the livers. Significantly more pseudocysts (total number) were located in the middle compared to the left (χ2=83.03 with Yates’ correction, p=0.00001) and right sections (χ2=112.29 with Yates’ correction, p=0.00001). However, the comparison of pseudocysts in the left and right parts of the liver was non-significant (p > 0.05). When comparing sections with the highest number of pseudocysts per each liver, it is evident that samples with highest number of pseudocysts in the middle part (n=38) were significantly different (χ2 with Yates correction=28.0629, p=0.00001) to the ones in right (n=5), and left part (n=7; χ2 with Yates correction=23.7657, p=0.00001).

**Table 2.** Odds of finding pseudocysts in liver sections compared to the middle section, and the left vs. right section.

	Red deer			Fallow deer		
	middle vs left	middle vs right	left vs right	middle vs left	middle vs right	left vs right
odds ratio	7.0467	10.0115	1.420	3.7063	3.8246	1.0319
95% CI	3.024-16.41	3.808-26.321	0.4401-4.5868	2.3529-5.8379	2.4227-6.0377	0.6313-1.6867
z statistic	4.525	4.671	0.587	5.651	5.759	0.125
significance	p=0.0001	p=0.0001	p=0.557	p=0.0001	p=0.0001	p =0.9003

Analysis of livers containing 10 or more pseudocysts in any of the sections showed that the highest number of pseudocysts was in the middle section in all samples. The odds of finding the highest number of pseudocysts in the middle section were 10 times higher compared to the right and 7 times higher compared to the left section (for both, p = 0.0001). Upon further analysis of the left and right sections, the highest number of pseudocysts was found in the left part of seven livers and the right part of six livers. A comparison of the left and right sections revealed an odds ratio of 1.4, indicating a 1.4 times higher chance that the left section would have more pseudocysts than the correct section. However, the difference was not statistically significant (p > 0.05).

### Fallow deer

As in the case of red deer, only positive livers were analysed and the highest total number of pseudocysts was found in the middle section of livers (n=95, $\bar{\text{x}}=1.38$, min=0; max=8; SD=1.3853), followed by the left (n=42; $\bar{\text{x}}=0.6$; min= 0; max=3; SD=0.8236), and right sections (n=41; $\bar{\text{x}}=0.5857$; min=0; max=5; SD=1.1098). On average, 53.37 % of all pseudocysts were located in the middle section, 23.59 % in the left, and 23.03 % in the right section. The results of descriptive statistics are shown in Table 1.

Table 2 presents a comparison between the middle and left/right sections of the liver. Significantly more pseudocysts (total number in the sample) were located in the middle part compared to the left (χ2=14.21 with Yates’ correction, p<0.001) and right sections (χ2=14.91 with Yates’ correction, p<0.001). The number of pseudocysts in the left and right sections was nearly identical.

The highest number of livers had a majority of pseudocysts located in the middle section (n = 36; 63.2 %), followed by the left (n = 11; 19.3 %) and right sections (n = 10; 17.5 %). The odds of finding pseudocysts in the middle section were 3.7 times higher than in the left section, and 3.8 times higher than in the right section.

For both red and fallow deer, there was no evident pattern in the localization of the pseudocysts, regardless of their number (severity of infection). Some livers had pseudocysts only in one section, which did not align with the expectation that pseudocysts would typically be located in the middle section.

## Discussion

Upon entering the host’s digestive system, the juvenile fluke emerges from the metacercariae, penetrates the intestinal wall, and travels along the ventral abdominal surface in search of the liver (Pybus, 2001). Occasionally, during abdominal migration in aberrant hosts, the juvenile fluke can mistakenly end up in other organs, such as the spleen, lungs, or kidneys (Conboy *et al*., 1991; Foreyt, 1996; Karamon *et al*., 2015; Stiles *et al*., 2021). According to previous studies on *F. hepatica*, only 50 % of juvenile flukes reach the liver (Montgomerie, 1928). However, this does not guarantee their survival, as a study by Foreyt and Todd (1976) showed that only 5 – 6 % of experimentally administered flukes reach sexual maturity. Flukes that successfully reach the liver will penetrate the Glisson’s capsule and enter the liver parenchyma (Králová-Hromadová *et al*., 2016). The next phase involves migration through the liver, where it feeds on liver tissue and blood. It is assumed that migration is also a mechanism by which the fluke can evade the host’s immune response (Meeusen *et al*., 1995). When the fluke completes its development, it ends the migration phase, and the organism can form a pseudocyst. Based on the observed migratory paths, it appears that the fluke migrates over a larger portion of the liver, and the initial period of migration does not necessarily predict the future localization of pseudocysts. Recent studies have shown that all three types of hosts can form pseudocysts (Králová-Hromadová *et al*., 2016; Demiaszkiewicz *et al*., 2018; Konjević *et al*., 2021).

This study reveals that in both wild definitive hosts, the majority of pseudocysts are formed in the middle section of the liver, which is significantly higher compared to the left and right sections. When comparing red deer and fallow deer, 73 % and 63.2 % of pseudocysts were located in the middle section of the liver, respectively. The odds of finding pseudocysts were higher for the middle section of the liver compared to the left and right sections. Also, the odds were higher in red deer, which is understandable since red deer in general harbour more pseudocysts than fallow deer (red vs. fallow deer: middle (mean number of pseudocysts) 4.58:1.36; left 2.24:0.6; right 1.93:0.59). When comparing the left and right sections of the livers, slightly higher numbers of pseudocysts were found in both species in the left section (red deer 16.54 % vs. 10.53 %; fallow deer 19.3 % vs. 17.5 %). Unlike the clear pattern of more pseudocysts in the middle section of the liver, the distribution of pseudocysts in the left and right sections showed no statistical differences. If we analyze only cases in which the most significant number of pseudocysts in the liver was located in the left or right section, it is more likely to find pseudocysts in the left part than in the right part. The localization of pseudocysts in the middle section of the liver can be explained by the fact that the majority of blood vessels are present in this area, since flukes feed on blood. The role of blood in the fluke diet is also visible through the production of iron-porphyrin, a pigment that is a by-product of feeding on blood (Campbell, 1961), and a pathognomonic sign of *F. magna* infection. Additionally, the main bile ducts are located in this section of the liver, which is important for the transmission of eggs into the environment.

This study demonstrated that pseudocyst localization cannot be used as a criterion for grading the severity of infection. Even though the majority of pseudocysts are located in the middle section of the liver, in some cases, more pseudocysts can be found in other sections. For instance, in red deer, 11 of 143 livers had the highest numbers of pseudocysts located in the left section. In fallow deer, 11 livers had the highest number of pseudocysts in the left section, and 10 in the right one. Also, the localization of the pseudocysts in lateral sections was almost identical.

Examining red deer livers containing 10 or more pseudocysts, the largest number of pseudocysts was found in the middle section, while the left and right sections contained nearly equal numbers of pseudocysts. A similar result, but with fewer pseudocysts in general, was found in fallow deer.

Given that the large American liver fluke *Fascioloides magna* is a non-native species in Europe, it is particularly important to monitor the events from miracidia to the formation of the pseudocyst in the liver of the final host. Monitoring these events can shed light on the development of the host-parasite association and potential adaptations, particularly at the levels of aberrant and dead-end hosts.
